# Differences in HPV-specific antibody Fc-effector functions following Gardasil® and Cervarix® vaccination

**DOI:** 10.1038/s41541-023-00628-8

**Published:** 2023-03-15

**Authors:** Vicky Roy, Wonyeong Jung, Caitlyn Linde, Emily Coates, Julie Ledgerwood, Pamela Costner, Galina Yamshchikov, Hendrik Streeck, Boris Juelg, Douglas A. Lauffenburger, Galit Alter

**Affiliations:** 1 Ragon Institute of MGH, MIT, and Harvard, Cambridge, MA USA; 2 Institute of Virology, University Hospital Bonn, Bonn, Germany; 3 Vaccine Research Center, National Institute of Allergy and Infectious Diseases, National Institutes of Health, Bethesda, MD USA; 4 Department of Biological Engineering, Massachusetts Institute of Technology, Cambridge, MA USA

**Keywords:** Protein vaccines, Tumour virus infections

## Abstract

Gardasil® (Merck) and Cervarix® (GlaxoSmithKline) both provide protection against infection with Human Papillomavirus 16 (HPV16) and Human Papillomavirus 18 (HPV18), that account for around 70% of cervical cancers. Both vaccines have been shown to induce high levels of neutralizing antibodies and are known to protect against progression beyond cervical intraepithelial neoplasia grade 2 (CIN2+), although Cervarix® has been linked to enhanced protection from progression. However, beyond the transmission-blocking activity of neutralizing antibodies against HPV, no clear correlate of protection has been defined that may explain persistent control and clearance elicited by HPV vaccines. Beyond blocking, antibodies contribute to antiviral activity via the recruitment of the cytotoxic and opsonophagocytic power of the immune system. Thus, here, we used systems serology to comprehensively profile Gardasil®- and Cervarix®- induced antibody subclass, isotype, Fc-receptor binding, and Fc-effector functions against the HPV16 and HPV18 major capsid protein (L1). Overall, both vaccines induced robust functional humoral immune responses against both HPV16 and HPV18. However, Cervarix® elicited higher IgG3 and antibody-dependent complement activating responses, and an overall more coordinated response between HPV16 and 18 compared to Gardasil®, potentially related to the distinct adjuvants delivered with the vaccines. Thus, these data point to robust Fc-effector functions induced by both Gardasil® and Cervarix®, albeit with enhanced coordination observed with Cervarix®, potentially underlying immunological correlates of post-infection control of HPV.

## Introduction

Human Papillomavirus is the most common sexually transmitted disease in the United States^1^. There are over 200 known strains of the virus, that cause a range of pathologies from benign condylomas to invasive malignant tumors^2^. In fact, more than 70% of cervical cancers in women are caused by HPV16 and HPV18 infections. HPV has also been identified as a major cause of vulval, anal, vaginal, penile, and oral cancers, establishing HPV as a major public health target^2–4^.

To date, three vaccines have been licensed to prevent HPV infection and thus HPV related malignancies: (1) a quadrivalent vaccine HPV6/11/16/18 VLP vaccine co-delivered with aluminum hydroxyphosphate sulfate (Gardasil®, 4vHPV, Merck), (2) a bivalent HPV vaccine co-administered with an AS04 adjuvant (Cervarix®, 2vHPV, Glaxo Smith Kline), and (3) most recently a nonavalent HPV vaccine against HPV6/11/16/18/31/33/45/52/58 co-administered with aluminum hydroxyphosphate sulfate (Gardasil9®, 9vHPV, Merck)^5–7^. All 3 vaccines provide protection against HPV types 16 and 18 that cause most HPV associated malignancies. Multiple efficacy trials have been conducted for both Cervarix® and Gardasil®^8–16^. Overall, both vaccines show robust protection against persistent infection from vaccine strains and good efficacy against progression to cervical intraepithelial neoplasia (CIN)^9,15,17^. Although both vaccines have been shown to induce high titers of neutralizing antibodies against HPV16/18 and some phylogenetically related strains, longitudinal studies have suggested that Gardasil®-induced antibodies may wane more rapidly compared to Cervarix®-induced responses^6,16,18,19,15^.

Beyond the sterilizing protection afforded by HPV vaccines, they have also been linked to protection against neoplastic progression^15,20–22^. Specifically, both vaccines are able to restrict the progression of CIN^15,20–22^, by blocking infection and potentially restricting the evolution of infection. Moreover, data from clinical trials have shown that although Gardasil® is effective at preventing progression to CIN2+ by HPV genotypes included in the vaccine, Cevarix® offers superior protection against CIN2+, even against phylogenetically related non-vaccine genotype infections^15^. While neutralizing antibodies are thought to play a central role in sterilizing protection^23,24^, cellular immune responses have been implicated in the therapeutic activity of experimental HPV vaccines targeting the E6 and E7 oncoproteins^25^.

Beyond neutralization, antibodies have been implicated in the control and clearance of several other viral infections via their capacity to direct the antiviral activity of all innate immune cells via Fc-receptors (FcR). For example, the ability of virus-specific antibodies to drive antibody-dependent cellular cytotoxicity (ADCC) has been implicated in the control of Influenza^26,27^, opsonophagocytosis has been linked to protection against Ebola virus^28^, and the ability of inducing both cytotoxicity and opsonophagocytosis have been linked to control of Human Immunodeficiency Virus (HIV)^29,30^. Moreover, emerging data suggest that HPV-specific IgG monoclonal antibodies may also provide protection against infection in an Fc-dependent manner. Specifically, reduced protection against infection was noted in studies where the Fc-section was removed or when the challenge was performed in an Fc-receptor knockout mouse^31^. Thus, these data argue for an important role for Fc-effector function in antibody-mediated protection against HPV. However, the Fc-effector profiles induced by Gardasil® and Cervarix® have not been comprehensively mapped, although emerging data point to higher levels of neutralizing antibodies^6,15^, T cell responses, B cell responses, and cytokine responses following Cervarix® vaccination compared to Gardasil® against common HPV16/18 strains^6,14,16,18,32^. Thus, here we profiled the HPV-specific humoral responses elicited by Gardasil® and Cervarix® in a cohort of 24 Women over 13 months. Differences in isotype, subclasses, Fc-receptor binding, and Fc-effector functions were compared across HPV16 major capsid protein (L1) and HPV18 L1 over time^14^. Both vaccines induced robust humoral immune responses, with similar isotype/subclass, Fc-receptor binding profiles, and Fc-effector functions. However, Cervarix® induced higher IgG3 titers and complement-fixing antibodies compared to Gardasil®. Moreover, Cervarix® induced a highly coordinated functional humoral immune response toward HPV18 and HPV16 L1, whereas Gardasil® induced a highly variegated functional humoral immune response to the 2 HPV genotypes. Collectively, these data highlight the robust evolution of functional humoral immune responses, with key immunologic differences across HPV vaccine platforms and formulations that may contribute to differential outcomes following HPV infection.

## Results

### Gardasil® and Cervarix® induce similar overall antibody Fc-profiles over time

**Fig. 1:**
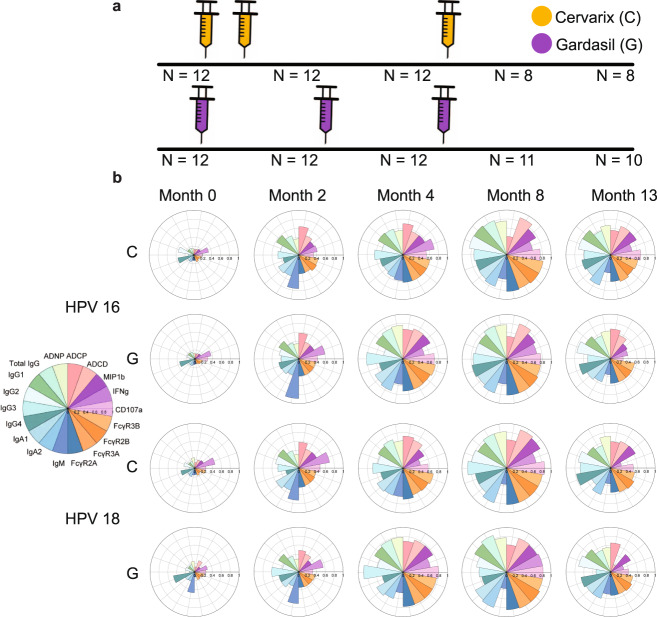
Vaccination schedule and overall immune responses. **a** Injection schedule for Cervarix® (orange) and Gardasil® (purple). **b** The polar bar plots show the median percentile of antibody features induced by Cervarix® (denoted by C) and Gardasil® (denoted by G) against HPV16 (top) and HPV18 (bottom) across time points. The size of the wedges depicts the magnitude of the value.

The Vaccine Research Center 902 cohort included 24 L1-specific T cell negative women ranging from 18–25 years old randomized at a 1:1 ratio to receive Cervarix® or Gardasil® (3 injections each with the Cervarix® group receiving the second dose 1 month ahead of the Gardasil® recipients) (Fig. 1a)^14^. We used samples from 12 Cervarix® recipients and 12 Gardasil® recipients for the analysis. Blood was collected at five timepoints (month 0, 2, 4, 8 and 13) for each participant. To begin, all samples were analyzed for isotypes and subclass levels against HPV 6/11/16/18 using Luminex. Following a single dose of Gardasil® and 2 doses of Cervarix®, comparable HPV16 and HPV18 L1-specific responses were observed at month 2 across isotype and subclass selection profiles (Fig. 1b). Although p-values calculated from two-sided Wilcoxon rank-sum test comparing the 2 vaccines were not statistically significant after multiple test corrections for all antibody features, the overall response induced by Gardasil® was lower at month 2, likely due to differences in the boosting schedule (Fig. 1a). Moreover, prior to the third dose, at month 4, the magnitude of the overall subclass/isotype specific response was slightly higher in individuals that received the Gardasil® vaccine compared to the Cervarix® vaccine. After the third dose, an expansion of both HPV16 and HPV18 L1-specific responses was observed across both vaccine groups, reaching peak immunogenicity at month 8. However, interestingly, at month 13, the final time-point, responses were slightly higher in individuals that received the Cervarix® vaccine. HPV6 and HPV11 L1-specific responses after vaccination were observed only for Gardasil® recipients (Supplementary Fig. 1), which is expected since only Gardasil® contains antigens for those strains.

### Subtle univariate differences emerge over time in antibody isotype and subclass profiles across Gardasil® and Cervarix® vaccine responses

**Fig. 2:**
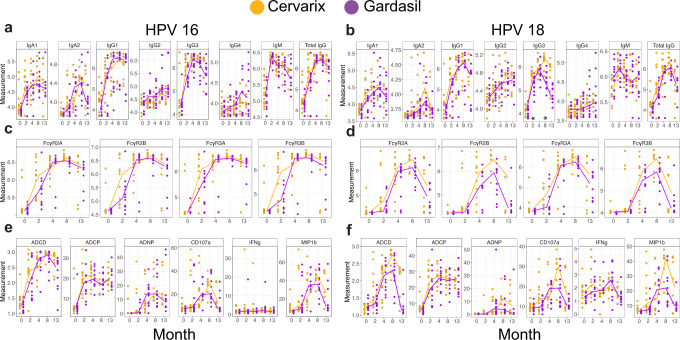
Overall response to vaccination over time. **a**–**f** Univariate plots showing levels of antibody titers (**a**, **b**), FcR binding (**c**, **d**), and Fc-effector functions (**e**, **f**). Fc-effector functions include antibody-dependent complement deposition (ADCD), antibody-dependent cellular phagocytosis (ADCP), antibody-dependent neutrophil phagocytosis (ADNP), and percentage of CD107a (CD107a), MIP-1β (MIP1b), and Interferon gamma (IFNg) positive cells. Measurement for each patient is denoted by dots across timepoints with running median for Cervarix® (orange) and Gardasil® (purple) against HPV16 (**a**, **c**, **e**) and HPV18 (**b**, **d**, **f**). Log10 of mean fluorescent intensity is used as a unit of measurement for antibody titers, FcR binding, and ADCD, whereas phagocytic score was used for ADCP and ADNP. For CD107a, MIP1b, IFNg, percentage of positive cells was used as a unit of measurement. Two-sided Wilcoxon rank-sum test was performed between the two vaccine groups. * indicates *p* < 0.05 after multiple-test correction using the Benjamini-Hochberg procedure for all comparisons shown in the figure.

To gain enhanced resolution of any specific differences across the 2 vaccine profiles, we next compared each feature individually over time across the vaccine groups (Fig. 2). First, comparison of antibody isotype/subclass selection pointed to similar total HPV16 L1-specific IgG (including IgG1, 2, 3, and 4) titers across the 2 vaccines (Fig. 2a). Conversely, total HPV18 L1-specific IgG titers were slightly higher over time in individuals that received Cervarix® compared to those that received Gardasil® (Fig. 2b, as previously reported^6,10,11^. All other isotypes and subclasses were remarkably similar across the 2 vaccine groups, with the exception of HPV18 L1-specific IgG3 (Fig. 2b) levels that were induced to a higher level in individuals that received the Cervarix® vaccine, reaching statistical significance calculated from two-sided Wilcoxon rank-sum test at months 2 and 8 after multiple test correction for all comparisons shown in Fig. 2.

### Altered Fc-receptor binding kinetics across the 2 vaccines

Next, we profiled the ability of the vaccines to induce antibodies able to interact with Fc-receptors, required to elicit Fc-effector functions^33,34^. Both vaccines induced antibodies able to interact with all 4 low-affinity IgG Fc-receptors, key to driving innate immune effector functions (Fig. 2c, d). Similar to subclass and isotype responses, Fc-receptor binding reached peak levels at month 8 for both vaccines. HPV16 L1-specific FcγR2a, FcγR3a, FcγR2b and FcγR3b binding antibodies emerged earlier in Cervarix® immunized individuals, and at slightly higher levels at peak immunogenicity in Cervarix® recipients (Fig. 2c), likely related to differences in vaccine regimens, but ultimately reached similar levels with similar kinetics across the 2 vaccines. Conversely, HPV18 L1-specific FcγR2a and FcγR3a binding responses were more similar across timepoints, but FcγR2b and FcγR3b binding antibodies diverged at months 8 and 13 following vaccination across the 2 vaccines (Fig. 2d), marked by enhanced Fc-receptor binding durability in Cervarix® immunized individuals compared to individuals that received Gardasil® hinting at a potential functional durability differences across the vaccines.

### Divergent durability in HPV18-effector function across Cervarix® and Gardasil®

Given the observed differences in the maintenance of FcR binding across the 2 vaccines, we probed the overall induction and durability of HPV16 and HPV18 L1-specific Fc effector functions across the 2 vaccine groups (Fig. 2e, f). Natural Killer cells (NK cell) activating antibodies were induced by both vaccines, although Cervarix® induced higher HPV16 and HPV18 L1 antibodies able to activate NK cell degranulation (CD107a) and chemokine MIP1b secretion at later timepoints. Both vaccines also induced antibody-dependent complement deposition (ADCD), antibody-dependent cellular phagocytosis (ADCP), and antibody-dependent neutrophil phagocytosis (ADNP), with a trend towards superior complement deposition at later timepoints in Cervarix® immunized individuals compared to those induced by Gardasil® (ADCD against HPV 16 L1 at month 13 (Fig. 2e) and ADCD against HPV18 L1 at month 8 (Fig. 2f) with *p* < 0.05 from two-sided Wilcoxon rank-sum test before multiple test correction).

### Differences in vaccine-induced HPV-specific humoral immune architecture across the vaccines

**Fig. 3:**
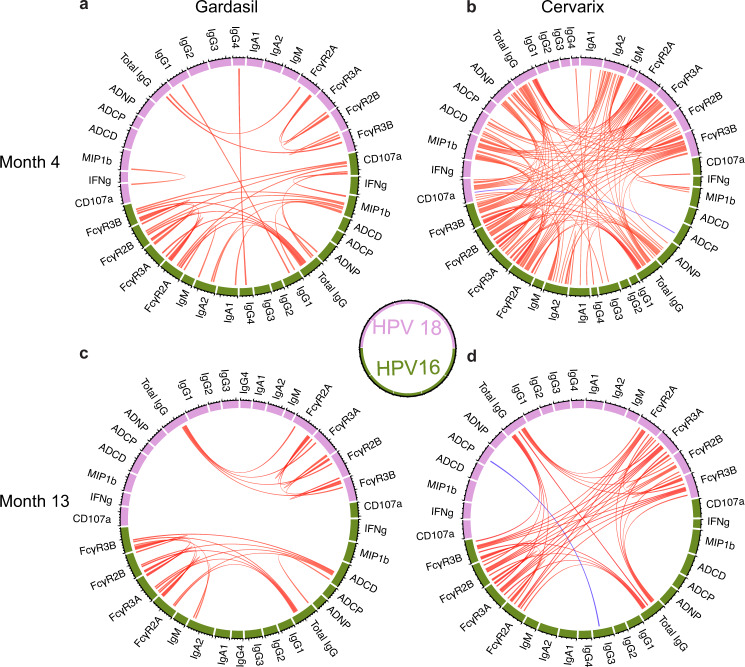
Correlation between immune responses at month 4 and 13. **a**–**d** Chord diagrams showing Spearman correlation (r) between features at month 4 and 13. Red and blue represent positive and negative correlation coefficient, respectively, and darker color represents a higher absolute value of the correlation coefficient. All shown correlations are those with |*r*| >0.7 and *p* < 0.01 after multiple test correction with the Benjamini-Hochberg procedure for all comparisons between features from each timepoint and each vaccine type. ADCD antibody-dependent complement deposition, ADNP antibody-dependent neutrophil phagocytosis, ADCP antibody-dependent cellular phagocytosis.

Differences in HPV18 immunity and to some degree in HPV16-immunity across the 2 vaccines pointed to potential differences in the overall immunodominance or balance of functional immunity across the strains. Emerging data suggest that Cervarix® provides more durable protection than Gardasil® against both HPV16 and HPV18^8,20,22,35–38^, although other studies have argued no difference in real-world HPV vaccine performance^39,40^. Thus, to further determine whether any additional difference exists across the vaccines, we next examined the coordination of HPV16 and HPV18 responses, by pair-wise correlations between functional humoral immune responses to both HPV strains at months 4 and 13 for Gardasil® and Cervarix® recipients (Fig. 3). For both vaccines, strong coordination was observed within vaccine strain, especially for total IgG and Fc-receptor binding, but cross-strain coordination was observed almost exclusively for Cervarix®. Moreover, at Month 4, Cervarix® showed strongly coordinated Fc-receptor and functional antibody responses, robustly driven by IgG titers across strains (Fig. 3b). Specifically, HPV-specific IgG levels continued to be tightly linked to Fc-antibody features across the strains. These data argue for unexpected differences in humoral coordination across the 2 vaccine platforms, marked by enhanced coordination and durability of cross-strain Fc-effector responses induced by Cervarix® compared to Gardasil®. Similar trends were also observed for the other timepoints (Supplementary Fig. 2).

### Distinct immunodominance patterns induced across the vaccines

**Fig. 4:**
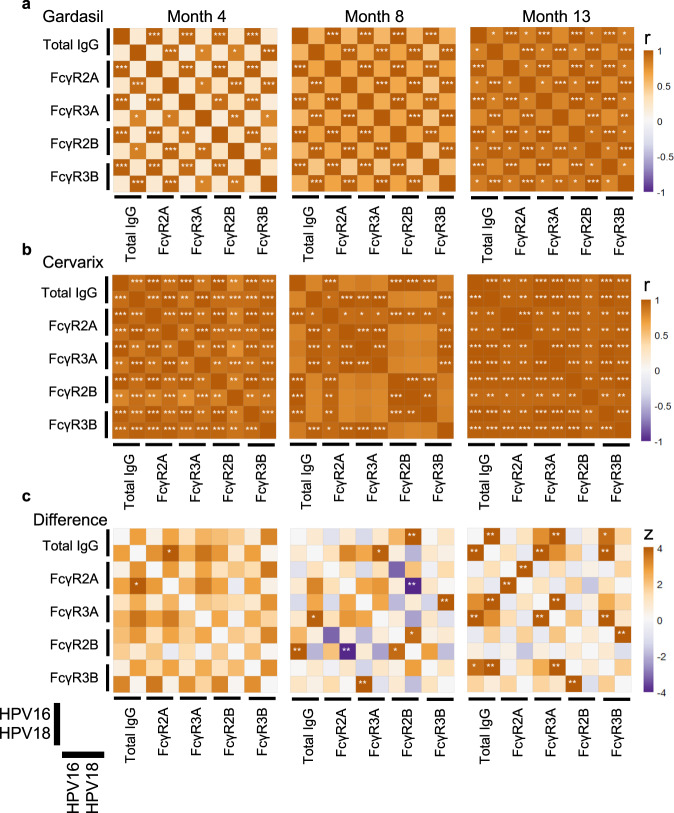
Differences across the two vaccine groups in correlation between immune responses to HPV genotypes. **a**, **b** Heatmaps showing Spearman correlation (*r*) between total IgG and Fc-receptor bindings in Gardasil® (**a**) and Cervarix® (**b**) recipients. Cervarix® group shows a more coordinated response across HPV 16 and 18. **c** Differential correlations (*z*) calculated using Fisher’s *r*-to-*z* transformation. Orange represents higher *r* for Cervarix® group, while purple represents higher *r* for Gardasil® group. *, **, and *** indicates 0.01 ≤ *p* < 0.05, 0.001 ≤ *p* < 0.01, and *p* < 0.001, respectively. *p*-values are multiple test-corrected by the Benjamini-Hochberg procedure for all comparisons between features from each timepoint.

To further quantify the coordination of HPV16 and HPV18 L1-specific immunity, we constructed a correlation heatmap using the dominant correlated features, namely total IgG and Fc-receptor binding levels (Fig. 4). Differences in the level of coordination between HPV16 and HPV18 L1-specific immunity were observable across Gardasil® and Cervarix®. At month 4, Gardasil® induced a highly coordinated HPV16 response, but more limited coordination within the HPV18 response or across strains (Fig. 4a). Conversely, at month 4, the Cervarix® response was highly coordinated both within and across strains (Fig. 4b). Moreover, at month 8 and 13, Gardasil® IgG titers were linked tightly to FcR binding within strains but exhibited limited cross-strain coordination compared to Cervarix® (Fig. 4a-c). On the other hand, at month 13, Cervarix® recipients showed an enhanced IgG-driven correlation with FcR binding within and across strains (Fig. 4b). Differences in correlation across the 2 vaccines became less pronounced by month 13 as Gardasil® recipients gained more cross-strain coordination, suggesting that additional doses may have shifted Gardasil® immunity towards a more balanced profile (Fig. 4a, c). Yet, some differences strongly persisted at month 13 across the 2 vaccine responses, most of which were related to cross-strain correlation differences, that were weaker for Gardasil® compared to Cervarix® induced profiles.

### Multivariate analysis distinguishes Gardasil® and Cervarix® vaccine profiles

**Fig. 5:**
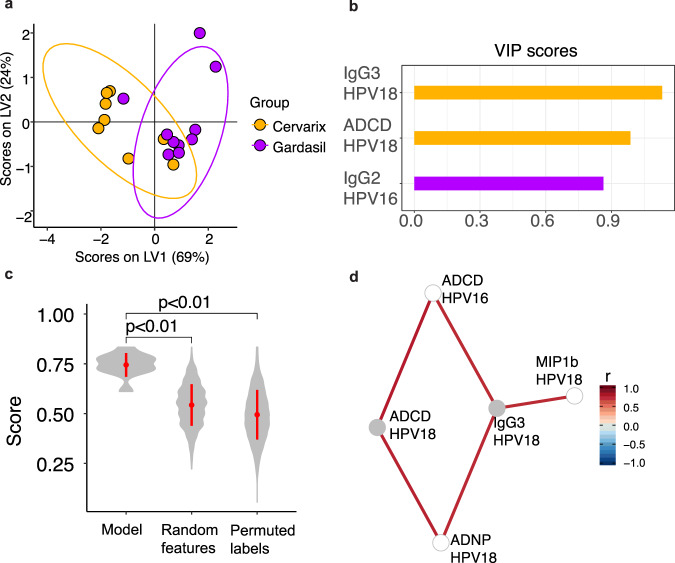
Multivariate analysis shows important features driving the separation of the two vaccine groups. **a**, **b** Samples at month 13 visualized using the first two latent variables (LV1 and LV2) from the Partial Least Square Discriminant Analysis (PLSDA) model (**a**) built with elastic net-selected features (**b**). Variable Importance in Projection (VIP) scores of the selected features are shown. *Q*^2^ values of the PLSDA model for LV1 and LV2 were 0.487 and −0.178, respectively. **c** Violin plots showing the distribution of 5-fold cross-validated accuracy scores from the original PLSDA model and alternate models. The accuracy of the original PLSDA model was significantly higher than the accuracy of the random features model and the permuted labels model. **d** Co-correlates of the elastic net-selected features enriched for the Cervarix® group. Features shown are those with |*r*| >0.7 and False Discovery Rate (FDR) < 0.05 after multiple test correction by the Benjamini-Hochberg procedure for all comparisons between features at month 13. ADCD antibody-dependent complement deposition ADNP antibody-dependent neutrophil phagocytosis.

Given the accumulating univariate differences observed across the 2 vaccine profiles, we finally aimed to determine whether a multivariate signature could be defined that could explain the greatest differences in the vaccine profiles induced by both vaccines (Fig. 5). Focusing on month 13, near-perfect separation was observed across the 2 vaccine profiles (Fig. 5a). Only 3 of the total 36 features were required to drive this separation, including Cervarix® levels of HPV18-L1-specific IgG3, HPV18 L1-specific ADCD activity, and Gardasil® levels of HPV16 L1-specific IgG2 (Fig. 5b). Importantly, comparison of model accuracy, compared to random data or permuted labels, highlighted the robustness of the model in separating out the 2 vaccine profiles (Fig. 5c). Given that the humoral immune response following vaccination was highly correlated, we next examined whether additional features were correlated with the model-selected features (Fig. 5d), which further highlighted differences across the 2 vaccine profiles. HPV18-L1-specific IgG3 were linked to enhanced levels of Antibody-dependent Neutrophil Phagocytosis (ADNP), Antibody-dependent Cellular Phagocytosis (ADCP), and MIP1b responses, pointing to higher overall functional antibodies following Cervarix® vaccination. Conversely, no additional features were significantly correlated to HPV16 L1-specific IgG2, suggesting a minimal biological role of this feature. Therefore, collectively, these data point to the presence of an expanded functional humoral immune response in individuals who received the Cervarix® vaccine, linked to the persistence of IgG3 responses, compared to responses induced by Gardasil®. Thus, the vaccines induced distinct Fc-effector profiles.

## Discussion

Human Papillomavirus (HPV) infection is the most common sexually transmitted diseases in the United States^1^. However, the introduction of HPV vaccines has significantly reduced rates of infection, especially the risk of HPV16 and HPV18 infection, that account for 70% of cervical cancers globally^3,4,17^. Yet, despite this extraordinary success, some differences have begun to emerge across the licensed HPV vaccines with respect to durability, as well as cross-protection against additional HPV strains^41–43^. However, while neutralizing antibodies represent a strong correlate of immunity, the precise mechanistic correlates of immunity against CIN progression remain incompletely defined due to the robust protection afforded by the vaccines proximal to vaccination. Yet, defining differences in immunity afforded by these vaccines could begin to highlight potential immunological mechanisms that could contribute to vaccine performance differences over time.

Among the licensed vaccines, Gardasil® and Gardasil9® are formulated with HPV16 and HPV18 L1 antigens in addition to two and seven, respectively, additional wart-causing HPV strains, driving a broader immunity to HPV viruses^5–7^. Conversely, Cervarix® includes only HPV16 and HPV18 L1 antigens, but has been shown to nonetheless drive a broad immunity to additional HPV variants^22^. It has been suggested that the AS04 adjuvant could be driving this broader immunity observed after Cervarix® vaccination^44^. Clinical trials have shown that Cervarix® offers superior protection toward phylogenetically related HPV strains that are not targeted by the vaccine compared to Gardasil® ^15^. Specifically, while both Gardasil® and Cervarix® were shown to offer 98% protection against the progression to CIN2 + caused by HPV16/18, protection against progression to CIN2+ by phylogenetically related HPV strains dropped to 62% for Cervarix® and 22% for Gardasil®. In addition, Cervarix® offered 93% protection against CIN3+ caused by phylogenetically related HPV genotypes compared to 43% for Gardasil®. Of note, the addition of five types of antigens in Garsasil9®, compared to Gardasil®, increased and broadened protection against infection and cross-protection against progression to CIN2+ that surpassed the levels induced by Cervarix®^15,20,40–43,45,46^. However, some evidence showed that Gardasil9® and Gardasil® both exhibited HPV18 L1-specific antibody waning^15^, which was observed to a lesser extent with Cervarix®^15,47^. Here, we observed differential functional IgG3 subclass selection, complement-inducing antibody function, and a significant immunodominance shift across the 2 vaccine platforms over time, which may provide some explanation for the immunity and protection differences observed with these HPV vaccines.

We observed robust strain-specific antibody profile coordination for Gardasil®, but poor cross-strain antibody response coordination early following vaccination. Conversely, Cervarix® induced robust within and cross-strain Fc-response profiles that were preserved for several months. One potential explanation for poor overall response coordination following Gardasil® vaccination may relate to differences in adjuvant capacity to break HPV-16 immunodominance at the time of vaccination^14,48^. Several studies have noted that some adjuvants are able to break immunodominance more effectively, driving epitope spreading, breadth, and durability^49,50^. In this context, several adjuvants have been shown to drive superior epitope targeting compared to alum^51–53^. Correlation analysis of IgG titers and Fc receptor binding for all four strains included in Gardasil® (Supplementary Fig. 3) highlighted that correlation across HPV16, HPV6 and HPV11 L1 appeared earlier (months 2, 4), while the correlation between HPV18 and HPV16 L1 appeared only after three doses (month 13). These data point to preferential presentation of HPV16/6/11, rather than HPV18. Moreover, skewing was observed in HPV16:HPV18 immunity following Gardasil® vaccination (Supplementary Fig. 4). This trend was not observed following Cervarix® vaccination, suggesting that the use of an alternate adjuvant, or fewer L1 antigens, in Cervarix® may somehow have shifted immunodominance, driving equivalent immunity to the different genotypes.

Both Gardasil® and Gardasil9® use aluminum hydroxyphosphate sulfate while Cervarix® uses AS04^5,7,16^. AS04, a TLR4 agonist combined with an aluminum salt, is known to be more efficient than aluminum salt alone at boosting the immune response to the vaccine. Studies of the AS04 adjuvant show that it can enhance the quality of the antigen-specific T cell response, cytokine release, and consequently, B cell response and antibodies, by activating antigen-presenting cells^44,54,55^. This may be key to driving a balanced immune response across L1 antigens. Interestingly, recent data from the influenza field^27,56,57^ suggest that adjuvants can indeed drive more effective affinity maturation of antibodies. Thus, particular adjuvants may have the capacity to breach immune response skewing, presumably from inflammation or other host genetic biases, that would be observed after non-adjuvanted protein vaccination.

Beyond immunodominance differences across the vaccines, our data point to additional Fc-profile differences across the vaccines, marked by elevated IgG3 titers and complement-fixing antibodies induced by Cervarix® compared to Gardasil®. As we have shown, at month 13 the main driver of separation between the two vaccines was IgG3 levels and ADCD activity against HPV18 L1. Although the difference was not as striking with HPV16 L1, IgG3 titers and ADCD activity still trended towards higher levels with Cervarix® vaccination at peak immunogenicity. This enhanced persistent functional humoral immune activity could also play a mechanistic role in HPV control and clearance post-acquisition and could account for lower rates of progression to CIN2+ with Cervarix® vaccination. Indeed, although it is generally thought that extracellular neutralization is the critical mechanism of blocking HPV infection following vaccination, emerging data point to a role for antibodies in degrading HPV16 pseudovirus via TRIM21-mediated proteasomal pathway. These new data point to a potential role for L1-specific antibodies in reducing persistent infection^58^. Moreover, because TRIM21 binding also triggers an inflammatory response and enhanced antigen-presentation, these antibody responses may trigger enhanced innate immune clearance^59–61^, and even enhanced presentation of viral peptides via MHC class I to CD8 + T cells^62^ that may lead to clearance of infected cells prior to full-blown viral production. However, whether the differences in antibody function observed here contribute to differential HPV clearance remains unclear, but points to differences across vaccine platforms that may inform future HPV therapeutic vaccine design strategies, boosting regimens, as well as vaccine design for other pathogens.

Moreover, in a multivariate analysis, IgG3 levels were tightly correlated with additional effector functions, pointing to the presence of a more functional (larger breadth of effector functions) humoral immune response with Cervarix® compared to Gardasil® vaccination. Given that IgG3 exhibits the overall highest affinity for Fc-receptors and C1q^63,64^ the persistent production of higher levels of IgG3, following Cervarix® immunization, may continue to promote enhanced Fc-effector functions and complement activity over time. These functionally enhanced IgG3 antibodies may have the capability to eliminate the virus and rapidly deploy the broad antiviral activity of the innate immune system, accelerating viral clearance and consequently controlling potential neoplastic growth and progression of disease. Conversely, although Gardasil® induced antibodies that may have the potential to block infection, lower protection against neoplastic progression may relate to the antibody’s limited ability to harness the innate immune response in addition to lower cross-neutralizing antibody responses^65^. Given our emerging appreciation for the role of adjuvants in shaping subclass selection^66,67^, via enhanced Th1 inflammatory cascades^68,69^, these data further point to the importance of the adjuvant in shaping the antiviral potency of the vaccine-induced immune response that may be key to limiting disease progression.

While clinical trials with Gardasil® and Cervarix® have both demonstrated remarkable protection against HPV infection, differences have begun to emerge in real-world vaccine efficacy and durability, particularly against additional HPV strains and protection against the progression of disease^15,41,70^. However, some studies have not observed differences across the vaccines^39^, highlighting an urgent need to define whether any differences exist across the vaccine immune profiles to address this controversial observation. The comprehensive, objective analysis provided by systems serology pointed to both univariate and multivariate differences in the vaccine-induced immune responses induced by Gardasil® and Cervarix®.

While durability differences have been assessed years after vaccination, it is uncertain whether the 13-month post-vaccination differences observed here contribute to long-term changes observed in larger, longer vaccine trials. Importantly, this study focused on participants’ range of age (18–25 years), distinct from the recommended age for vaccination (9–12 years) that may represent a limitation of the study. Moreover, this study used insect derived VLPs, which is what is used in the formulation of Cervarix®. Gardasil®, on the other hand, is made with yeast-derived VLPs. This introduces a potential bias where the antibodies produced by Cervarix® vaccinees could have a higher affinity to the insect-derived VLPs used in our assays than antibodies from Gardasil vaccinees®. Insect-derived proteins possess distinct glycosylation profiles, that are less complex than those found in humans^71,72^. Conversely, yeast glycans tend to be larger high mannosylated structures^73,74^. However, while L1 is traditionally thought to be pauci-glycosylated^75^, emerging data suggest that alterations in n-linked glycosylation may represent a key HPV diversification^76^. Thus, the use of glycosylated HPV in this study may be important to capture antibody responses to this evasive biology. Additionally, we did not look at FcγRI binding and only focused on the low affinity FcγRs (see methods section for more details). Lastly, while Gardasil® induced robust persisting responses to HPV16^15,40^, the persistence of the HPV18 response is controversial. Multiple studies have shown a waning of HPV-18 titers over time through ELISA and neutralization assay^6,14,19,41^, but other studies using competitive Luminex immune assays (cLIA) have not observed this waning^39,40^. The reason behind these differences remains unclear and will need to be examined further. Despite this controversy, it is clear that Cervarix® titers to both HPV16 and 18 remained detectable for nearly a decade following vaccination^45^. Thus, while our study highlighted significant differences in antibody profiles induced by Gardasil® and Cervarix® at peak immunogenicity, current and future studies on vaccine durability, in populations where some breakthrough progression may occur, are urgently needed to resolve this controversy and help guide the use of these and future vaccines aimed at providing protection against HPV.

## Methods

### Subjects

The VRC-900 trial (#NCT01132859) included 24 women ranging from 18–25 years old randomized at a 1:1 ratio to receiving Cervarix® or Gardasil® (3 injections each). Samples from 24 women, 12 Cervarix® recipients and 12 Gardasil® recipients, were used for the analysis. Blood was collected at five timepoints (month 0, 2, 4, 8 and 13) for each participant. No subjects had detectable HPV L1 specific CD4 + T cell response prior to vaccination^14^. Due to participants who dropped out during the study, there were 11 Gardasil® and 8 Cervarix® recipients at month 8 and there were 10 Gardasil® and 8 Cervarix® recipients at month 13. The samples were collected under the Vaccine Research Center’s (VRC), National Institute of Allergy and Infectious Diseases (NIAID), National Institutes of Health protocol VRC 900 (NCT01132859) in compliance with the NIH Institutional Review Board (IRB) approved protocol and procedures. All subjects met protocol eligibility criteria and agreed to participate in the study by signing the NIH IRB approved informed written consent. Research studies with these samples were conducted by protecting the rights and privacy of the study participants.

### Antigens

Insect-cell derived Virus-like particles (VLP) from these sources were used in this study:

Human Papilloma Virus type 6 (HPV6) L1 protein (VLP) (Sino Biological)

Human Papilloma Virus type 11 (HPV11) L1 protein (VLP) (Sino Biological)

Human Papilloma Virus type 16 (HPV16) L1 protein (VLP) (Sino Biological)

Human Papilloma Virus type 18 (HPV18) L1 protein (VLP) (Sino Biological)

Glycosylation in insect-cell is thought to reflect the low complexity glycans variations found on L1 proteins.

### Luminex isotyping and Fc array

Antigen-specific antibody subclass isotypes and FcγR binding were analyzed by Luminex multiplexing. The antigens were coupled to magnetic Luminex beads by carbodiimide-NHS ester coupling with an individual region per antigen. Coupled beads were incubated with different plasma dilutions for 2 h at room temperature in 384-well plates (Greiner Bio-One). Unbound antibodies were washed away, and subclasses and isotypes were detected with a respective phycoerythrin (PE)-conjugated antibody at 0.65 µg/ml (SouthernBiotech; anti-IgG 9040-09, anti-IgG1 #9052-09, anti-IgG2 #9060-09, anti-IgG3 #9210-09, anti-IgG4 #9200-09, anti-IgM #9020-09, anti-IgA1 # 9130-09, anti-IgA2 # 9140-09). For the FcγR binding, a respective PE–streptavidin (1:250 dilution) (Agilent Technologies #PJ31S-5) coupled recombinant and biotinylated human FcγR protein (1 µg/ml) was used as a secondary probe. After 1-h incubation, excessive secondary reagent was washed away, and the relative antibody concentration per antigen was determined on an iQue Screener (Intellicyt).

We tested FcγR2A, FcγR3A, FcγR2B and FcγR3B as they are low-affinity receptors and are recognized as key modulatory targets for optimizing vaccine-induced antibodies or therapeutics^34,77–79^. We did not include high-affinity FcγRI as there is no evidence that it shapes antibody effector function in vivo or ex vivo. The receptor has been shown to enhance neutralization of HIV-1 in artificial cell-line based systems, by trapping viruses outside of cells^80^, but there is no evidence that this receptor tunes the ability of antibodies to interact with the receptors that are involved in driving opsonophagocytic, cytotoxic, or immunomodulatory functions. Specifically, because FcγRI is a high-affinity receptor, it is rapidly saturated with serum IgGs^79,81^, providing little nuanced functional programming. Moreover, as a high-affinity receptor, there is limited evidence that Fc-variation shapes FcγRI binding. Thus, it is unclear how vaccines would tune a high-affinity interaction more broadly.

### Antibody-dependent cellular phagocytosis (ADCP) assay

The ADCP assay was adapted from Ackerman et al. 2011^82^. Briefly, antigen was biotinylated using sulfo-NHS LC-LC biotin, coupled to yellow-green fluorescent Neutravidin 1 μm beads (Invitrogen, #F8776) for 2 h at 37 ˚C and washed two times in 0.1% bovine serum albumin (BSA) in phosphate buffer saline (PBS). 10 μL/well of coupled beads were added to 96-well plates with 10 μL/well of diluted sample for 2 h at 37˚C to form immune complexes. After incubation, the immune complexes were spun down and the supernatant was removed. THP-1 cells were added at a concentration of 2.5 × 10^4^ cells/well and incubated for 18 h at 37 ˚C. After incubation, the plates were spun down, the supernatant was removed, and cells were fixed with 4% paraformaldehyde (PFA) for 10 min. Fluorescence was acquired with a Stratedigm 1300EXi cytometer. Phagocytic score was calculated using the following formula: (percentage of fluorescent+ cells) * (the geometric mean fluorescence intensity (gMFI) of the fluorescent+ cells)/10,000.

### Antibody-dependent neutrophil phagocytosis (ADNP) assay

The ADNP assay was adapted from Karsten et al.^83^. Antigens were coupled to beads and immune complexes were formed as described for ADCP. Neutrophils were isolated from fresh whole Acid Citrate Dextrose (ACD) blood using EasySep Direct Human Neutrophil Isolation kit (Stem Cell,#19666), resuspended in R10, and added to plates at a concentration of 5 × 10^4^ cells/well. The plates were incubated for 30 min at 37 ˚C. The neutrophil marker CD66b (Pacific Blue conjugated anti-CD66b; BioLegend, #305112) was used to stain cells (1:100 dilution). Cells were fixed for 10 min in 4% PFA. Fluorescence was acquired with a Stratedigm 1300EXi cytometer and phagocytic score was calculated as described for ADCP.

### Antibody-dependent complement deposition (ADCD) assay

The ADCD assay was adapted from Fischinger et al.^84^. Antigen was coupled to non-fluorescent Neutravidin 1 μm beads (Invitrogen, #F8777) as described for ADCP. Immune complexes were formed by incubating 10 μL of coupled beads with 10 μL of diluted sample for 2 h at 37 ˚C. Plates were spun down, and immune complexes were washed with PBS. Lyophilized guinea pig complement (Cedarlane, #CL4051) was resuspended in cold water, diluted in Gelatin Veronal Buffer, Boston BioProducts, IBB-290X) and added to the immune complexes. The plates were incubated for 50 min at 37 ˚C and the reaction was stopped by washing the plates twice with 15 mM EDTA in PBS. To detect complement deposition, plates were incubated with Fluorescein-conjugated goat anti-guinea pig complement C3 (1:50 diluion) (MP Biomedicals, #0855385) for 15 min in the dark. Fluorescence was acquired with a Stratedigm 1300EXi cytometer.

### Antibody-dependent Natural Killer cell activation (ADNKA)

ELISA plates were coated with antigen at 300 ng/well and incubated for 2 h at 37 ˚C. Plates were blocked with 5% BSA in PBS overnight at 4 ˚C. The next day, 100 μL of diluted sample were added to the plates. Plates were incubated for 2 h at 37 ˚C to form immune complexes. During the incubation, human NK cells were isolated from buffy coats using RosetteSep NK cell enrichment kit (StemCell Technologies #15065) and Ficoll separation. After the incubation, NK cells were added to the plates at 5 × 10^4^ cells/well in R10 supplemented with anti-CD107a PE-Cy5 (1:80 dilution), GolgiStop and Brefeldin A (BD Biosciences,#554724, #555802, Sigma #B7651). Plates were incubated for 5 h at 37˚C. Following the incubation, NK cells were stained for surface markers with anti-CD56 PE-Cy7, anti-CD16 APC-Cy7 and anti-CD3 Pacific Blue (1:200 dilution) (BD Biosciences, #557747, #557758, #558124). NK cells were fixed and permeabilized with Fix&Perm cell permeabilization kit (Invitrogen). Cells were incubated with anti-MIP1β PE (1:200 dilution) and anti-IFNγ FITC (1:80 dilution) (BD Biosciences, #550078, #340449) to stain for intracellular markers. Cells were acquired on a Stratedigm 1300EXi cytometer.

### Statistical analysis overview

Except for the polar bar plots which were calculated and drawn with Python3, statistical analysis was performed with R (version 4.1.0). Before all statistical analysis, antibody titers, Fc-receptor binding, and ADCD data were log-transformed using log10. For multivariate analysis, data were z-scored after the log-transformation. Missing datapoints (1 datapoint out of 109 datapoints for HPV16-CD107, HPV16-IFNg, and HPV16-MIP1b and 8 datapoints out of 109 datapoints for HPV18-FcγR2B) were replaced by the median of the values from the other samples within each timepoint and each vaccine group. For all statistical tests, multiple test correction was done by Benjamini-Hochberg procedure^85^.

### Univariate analysis

Univariate analysis was performed comparing each feature from the Gardasil® and the Cervarix® groups at each time point. Two-sided Wilcoxon rank-sum test was done to determine if a significant difference exists between the two groups. *p*-values were multiple test-corrected using Benjamini-Hochberg procedure for all comparisons.

### Correlation analysis

Within each vaccine group at each time point, Spearman correlation between features and p-values were calculated, followed by multiple test corrections by the Benjamini-Hochberg procedure for all comparisons. To compare correlation coefficients from the two vaccine groups, we calculated differential correlation which can be calculated by first transforming the Pearson correlation coefficients (*r*) from each vaccine group to Z_*r*_ using Fisher’s Z-transformation^86^:

1 $$Z_r = 0.5\ln \frac{{1 + r}}{{1 - r}};\,{{{\mathrm{SE}}}} = \left( {N - 3} \right)^{ - 1/2}$$

SE is the standard error of *Z*_*r*_ and *N* is the number of correlations in each vaccine group. We used Spearman coefficient instead of Pearson coefficient as *r*, which was justified in^86^. Then, *Z*-test statistic was calculated by an equation:

2 $$z = \frac{{Z_{r,\,{{{\mathrm{Cervarix}}}}} - Z_{r,\,{{{\mathrm{Gardasil}}}}}}}{{\left[ {\left( {N - 3} \right)^{ - 1} + \left( {N - 3} \right)^{ - 1}} \right]^{1/2}}}$$

Higher *z* indicates a higher Spearman correlation coefficient calculated from the Cervarix® group, while lower *z* indicates a higher Spearman correlation calculated from the Gardasil® group. *z* closer to zero indicates that the correlation coefficients from the two vaccine groups are similar. *p*-values of *z* were multiple-test corrected by the Benjamini-Hochberg procedure for all comparisons within a vaccine group.

### Feature selection and classification

To find features that best classify the vaccine groups, we first selected features using logistic regression and elastic net regularization^87^, which were done 100 times. Features that were selected from more than 90 rounds were finally selected. Then, with the selected features, we built a model that classifies the two vaccine groups using Partial Least Square Discriminant Analysis (PLSDA)^88^. The Variable Importance in Projection (VIP) score of each selected feature, which explains the feature’s loading weights and the variance of the response (i.e. the vaccine group) explained by the feature, was calculated. For validation of the classification model, alternate models were built 100 times with either permuted response labels or randomly selected features. Then, the average accuracy of the original model from five-fold cross-validation was compared with those of alternate models. p-value for the comparison was calculated as the number of each alternate model that has higher accuracy than the average accuracy of the original model, divided by the number of each alternate model (i.e. 100). This cross-validation procedure was repeated 10 times and the median of *p*-values from 10 repetitions was used as a final *p*-value for the model comparison. A co-correlate network was built by calculating correlations between the selected features and all features and by showing the features that have a correlation with |*r*| >0.7 and FDR < 0.05 after multiple test correction by the Benjamini-Hochberg procedure for all comparisons. Elastic net and PLSDA were implemented using “glmnet” and “ropls” packages in R, and co-correlate network was generated by an R package “network.”

### Notes

Cervarix® is a registered trademark of GSK plc. Gardasil® is a registered trademark of Merck and Co., Inc.

### Reporting summary

Further information on research design is available in the Nature Research Reporting Summary linked to this article.

## Supplementary information

supplementary material

REPORTING SUMMARY

## Data Availability

Data and code used in the study are available from the corresponding author upon request.
