# Supplementary material for: Differences in HPV-specific antibody Fc-effector functions following Gardasil® and Cervarix® vaccination
Source: NPJ Vaccines. 2023 Mar 15;8:39. doi: 10.1038/s41541-023-00628-8 (PMC10017795; doi:10.1038/s41541-023-00628-8)
Supplement: Supplementary file 1 — supplementary material [file 41541_2023_628_MOESM1_ESM.pdf]

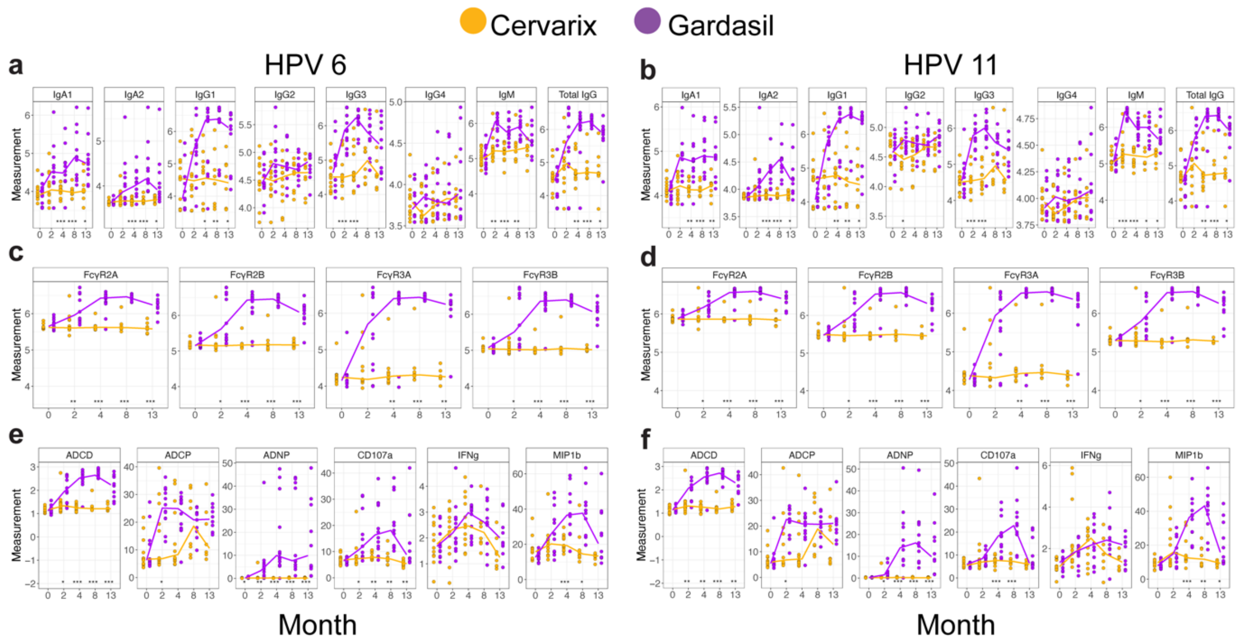

Supplementary Figure 1. Overall response to vaccination over time for HPV6 and HPV11. a-f. Univariate plots showing a level of antibody titers (a, b), FcR binding (c, d), and Fc-effector functions (e, f). Fc-effector functions include antibody-dependent complement deposition (ADCD), antibody-dependent cellular phagocytosis (ADCP), antibody-dependent neutrophil phagocytosis (ADNP), and percentage of CD107a (CD107a), MIP-1β(MIP1b), and Interferron gamma (IFNg) positive cells. Measurement for each patient is denoted by dots across timepoints with running median for Cervarix® (orange) and Gardasil® (purple) against HPV6 (a, c, e) and HPV11 (b, d, f). Log10 of mean fluorescent intensity is used as a unit of measurement for antibody titers, FcR binding, and ADCD, whereas phagoscore was used for ADCP and ADNP. For CD107a, MIPb, IFNg, percentage of positive cells was used as a unit of measurement. Two-sided Wilcoxon rank-sum tests were performed between the two vaccine groups. *, **, and *** indicates $0.01 \leq p < 0.05$, $0.001 \leq p < 0.01$, and $p < 0.001$, respectively. P-values are multiple test-corrected by the Benjamini-Hochberg procedure for all features shown in the figure.

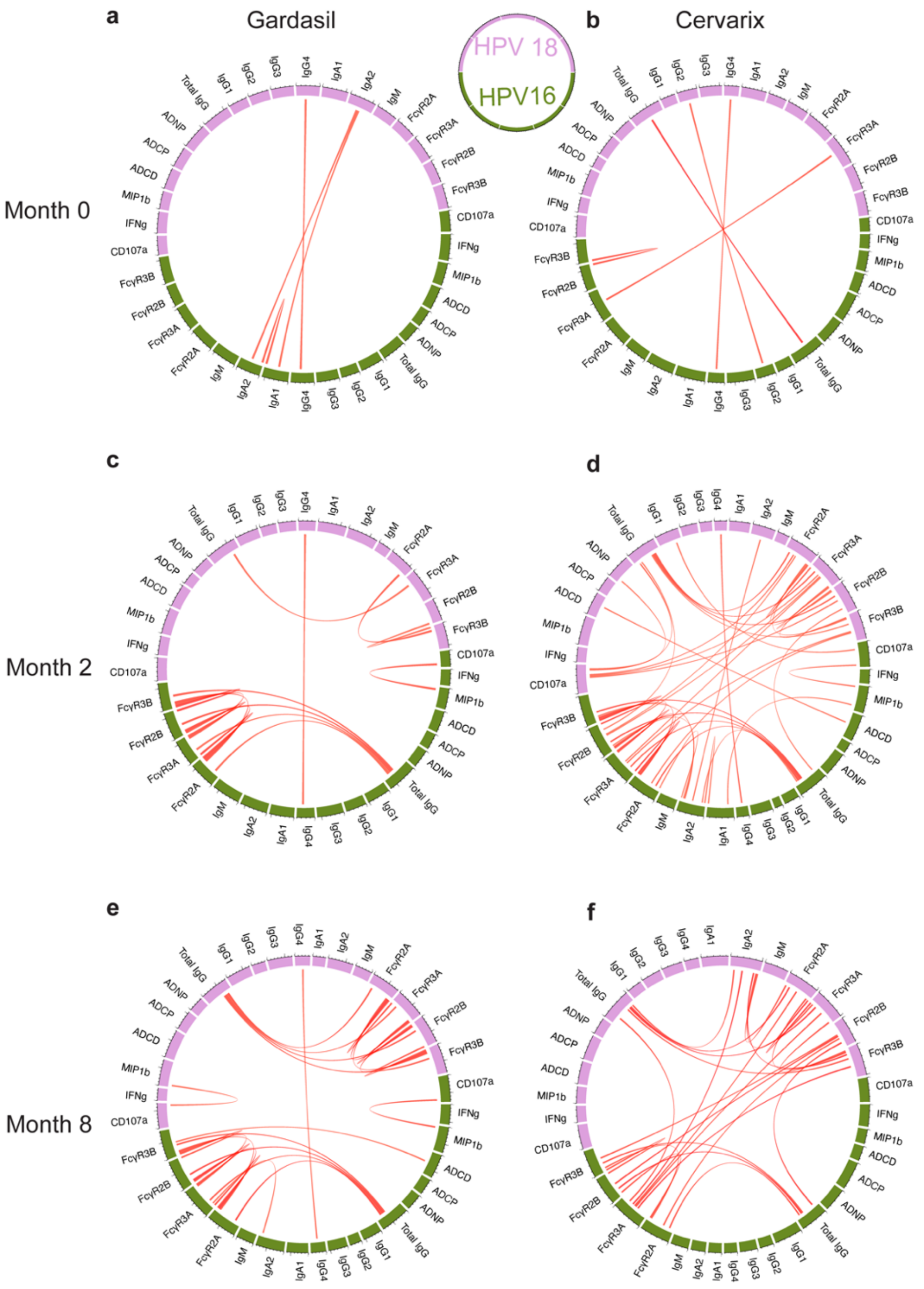

Supplementary Figure 2. Correlation between immune responses at month 0, 2, and 8. a-f. Chord diagrams showing Spearman correlation (r) between features at month 0, 2, and 8. Red and blue represent positive and negative correlation coefficient, respectively, and darker color represents a higher absolute value of correlation coefficient. All shown correlations are those with |r|>0.7 and p <0.01 after multiple test correction with the Benjamini-Hochberg procedure for all comparisons between features from each timepoint and each vaccine type. ADCD: Antibody-Dependent Complement Deposition ADNP: Antibody-Dependent Neutrophil Phagocytosis ADCP: Antibody-Dependent Cellular Phagocytosis.

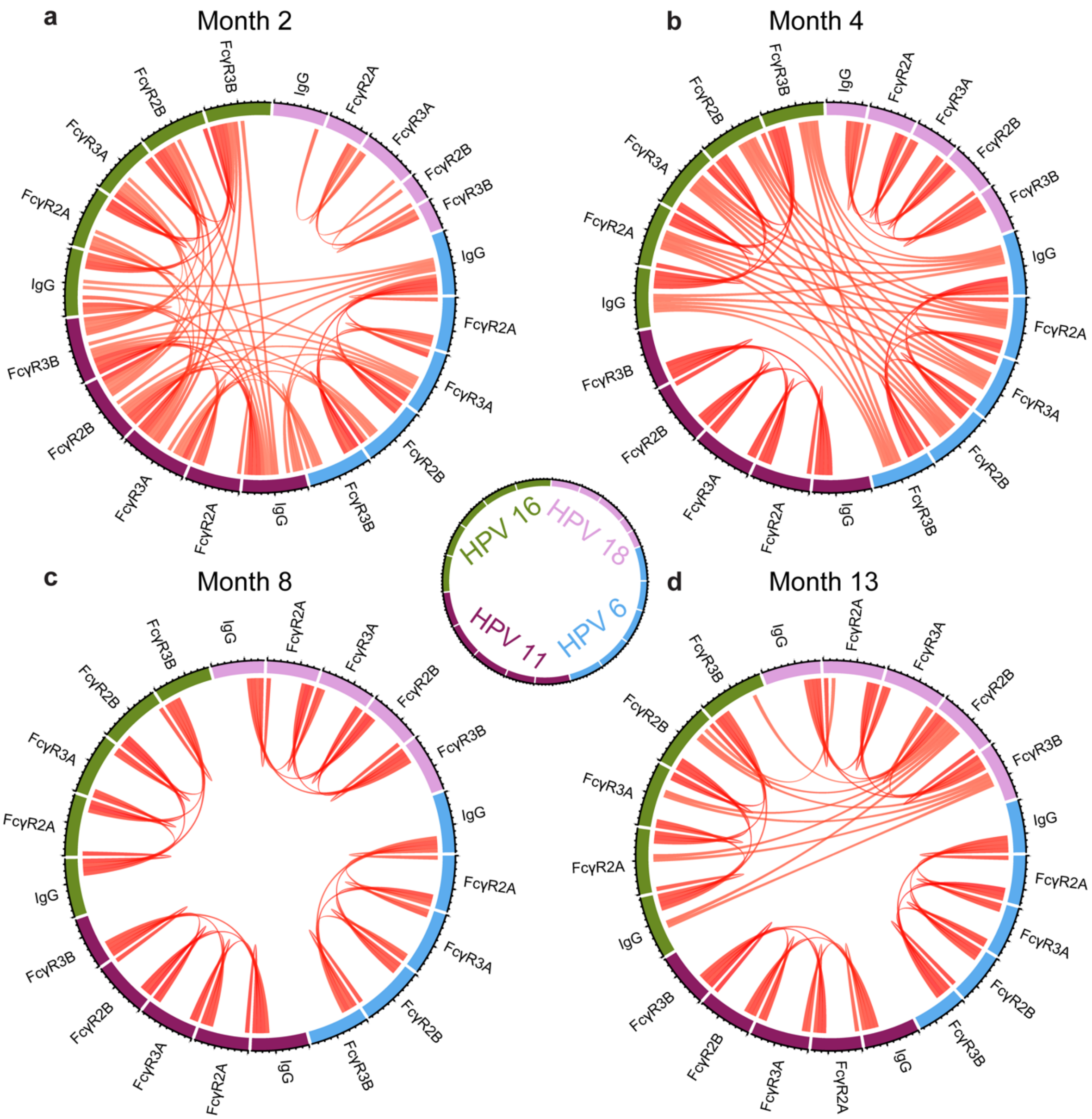

Supplementary Figure 3. Correlation between immune responses to HPV genotypes in Gardasil® recipients. a-d. Chord diagrams showing Spearman correlation between total IgG (denoted by IgG) and Fc-receptor bindings for the Gardasil® group across all HPV genotypes at month 2, 4, 8, and 13. At month 2, correlation between HPV6 and 11 and between HPV 11 and 16 appears. At month 4, correlation between HPV 6 and 16 appears. At month 13, correlation between HPV 16 and 18 appears. All shown correlations are those with |r|>0.7 and p <0.01 after multiple test correction with the Benjamini-Hochberg procedure for all comparisons between features from each timepoint.

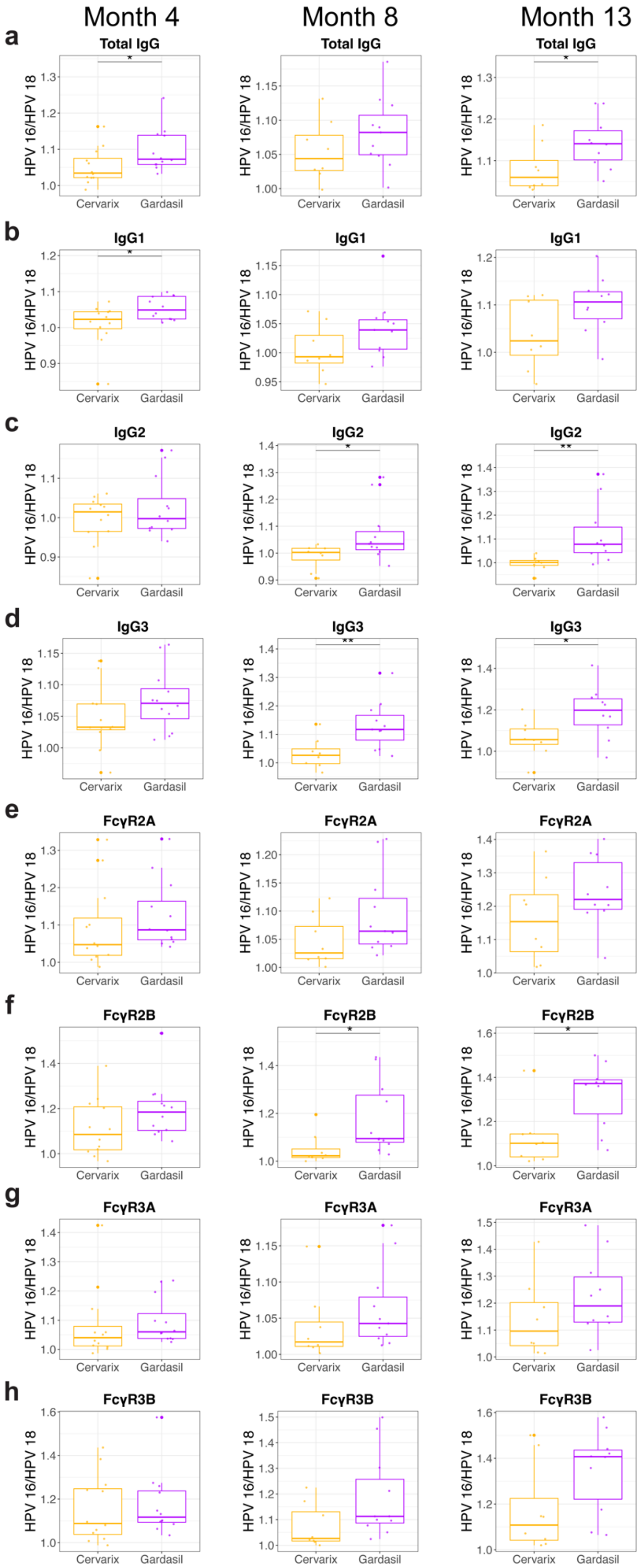

Month 4
Month 8
Month 13
a
Total IgG
b
IgG1
c
IgG2
d
IgG3
e
FcγR2A
f
FcγR2B
g
FcγR3A
h
FcγR3B
HPV 16/HPV 18
Cervarix
Gardasil

Supplementary Figure 4. Ratio between HPV16 L1-specific response and HPV 18 L1-specific response. a-h. The ratio between total IgG (a), IgG1 (b), IgG2 (c), IgG3 (d), FcγR2A binding (e), FcγR2B binding (f), FcγR3A binding (g), and FcγR3B binding (h), at month 4, 8, and 13. Log 10 of data was used to calculate the ratio. A ratio closer to 1 indicates a more balanced immune response across the HPV genotypes. Dots indicate the ratio from individual participants. In individual boxes and whiskers, the upper part of the box covers the third quartile to the median, while the lower part of the box covers the median to the first quartile. The upper whisker covers the maximum value except for outliers to the third quartile, while the lower whisker covers the first quartile to the minimum value except for outliers. Dots that are located above or below the whiskers are outliers that are defined as ratio > third quartile +1.5*(third quartile – first quartile) or ratio < first quartile - 1.5*(third quartile – first quartile), respectively. *, **, and *** indicates $0.01 \leq p<0.05$, $0.001 \leq p<0.01$, and $p<0.001$, respectively, before multiple-test correction. P-values were calculated from two-sided Wilcoxon rank-sum test. No features were statistically significant after multiple test correction with the Benjamini-Hochberg procedure for all features from each timepoint.

Gating Strategy

ADNP

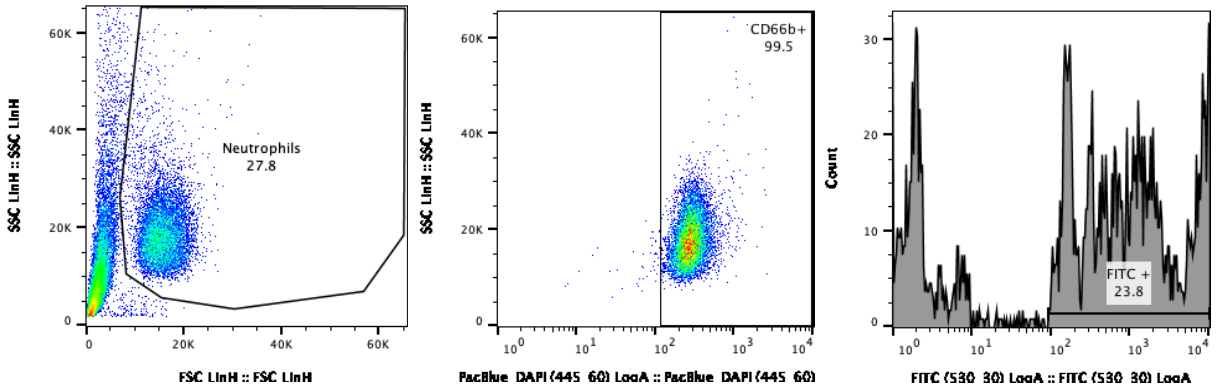

ADCP

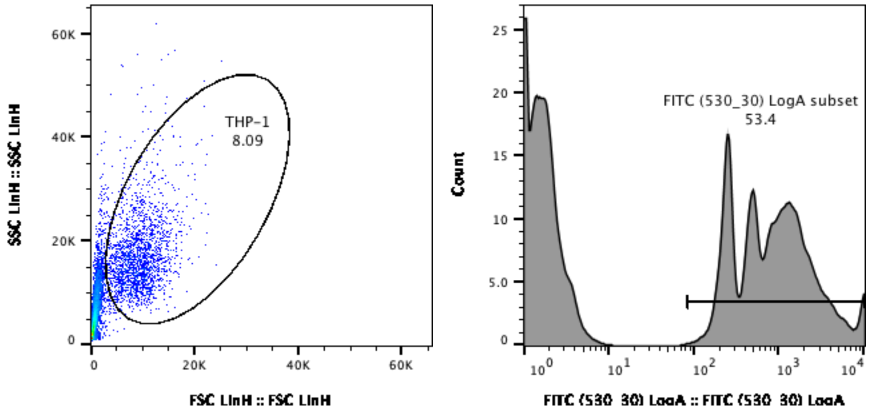

ADCD

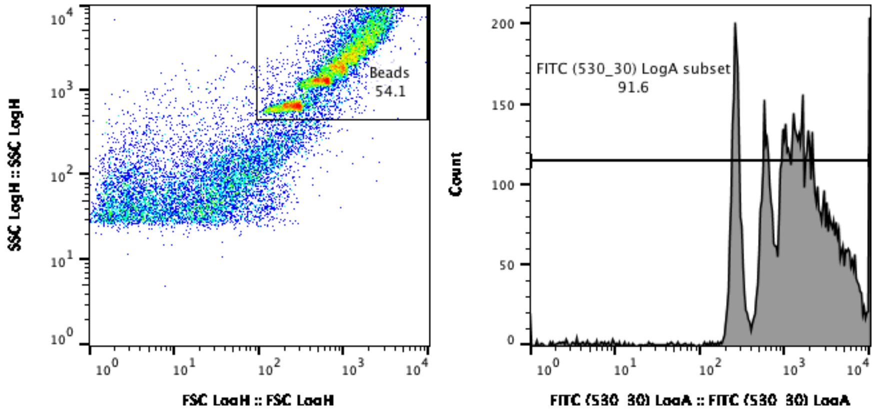

ADNKA

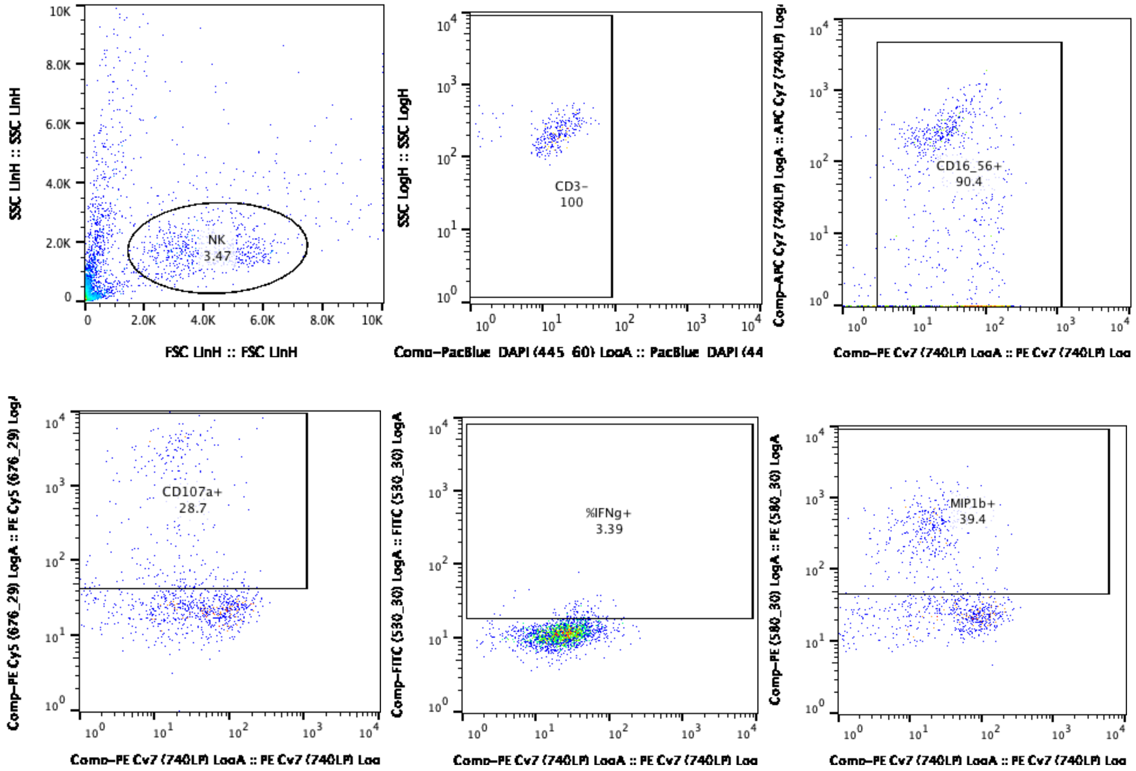
NK
3.47
SSC LinH :: SSC LinH
FSC LinH :: FSC LinH
CD3-
100
SSC LogH :: SSC LogH
CD16_56+
90.4
CD107a+
28.7
%IFNg+
3.39
MIP1b+
39.4
