# Supplementary material for: Differences in HPV-specific antibody Fc-effector functions following Gardasil® and Cervarix® vaccination
Source: NPJ Vaccines. 2023 Mar 15;8:39. doi: 10.1038/s41541-023-00628-8 (PMC10017795; doi:10.1038/s41541-023-00628-8)
Supplement: Supplementary file 2 — REPORTING SUMMARY [file 41541_2023_628_MOESM2_ESM.pdf]

nature portfolio

Corresponding author(s): Galit Alter

Last updated by author(s): May 29, 2022

Reporting Summary

Nature Portfolio wishes to improve the reproducibility of the work that we publish. This form provides structure for consistency and transparency in reporting. For further information on Nature Portfolio policies, see our Editorial Policies and the Editorial Policy Checklist.

Statistics

For all statistical analyses, confirm that the following items are present in the figure legend, table legend, main text, or Methods section.

n/a	Confirmed	
☐	☒	The exact sample size (*n*) for each experimental group/condition, given as a discrete number and unit of measurement
☐	☒	A statement on whether measurements were taken from distinct samples or whether the same sample was measured repeatedly
☐	☒	The statistical test(s) used AND whether they are one- or two-sided<br>*Only common tests should be described solely by name; describe more complex techniques in the Methods section.*
☐	☒	A description of all covariates tested
☐	☒	A description of any assumptions or corrections, such as tests of normality and adjustment for multiple comparisons
☐	☒	A full description of the statistical parameters including central tendency (e.g. means) or other basic estimates (e.g. regression coefficient) AND variation (e.g. standard deviation) or associated estimates of uncertainty (e.g. confidence intervals)
☐	☒	For null hypothesis testing, the test statistic (e.g. *F*, *t*, *r*) with confidence intervals, effect sizes, degrees of freedom and *P* value noted<br>*Give P values as exact values whenever suitable.*
☒	☐	For Bayesian analysis, information on the choice of priors and Markov chain Monte Carlo settings
☒	☐	For hierarchical and complex designs, identification of the appropriate level for tests and full reporting of outcomes
☐	☒	Estimates of effect sizes (e.g. Cohen's *d*, Pearson's *r*), indicating how they were calculated

Our web collection on statistics for biologists contains articles on many of the points above.

Software and code

Policy information about availability of computer code

Data collection	CellCapTure 4 (Stratedigm), Flowjo 10 (BD), Forecyt 9 (Sartorius), Excel 16
Data analysis	Python 3.0 (stats package from scipy, matplotlib), R 4.1.0 and R Studio version 1.4.1717 (R packages ropls, Hmisc, and tidyverse)

For manuscripts utilizing custom algorithms or software that are central to the research but not yet described in published literature, software must be made available to editors and reviewers. We strongly encourage code deposition in a community repository (e.g. GitHub). See the Nature Portfolio guidelines for submitting code & software for further information.

Data

Policy information about availability of data

All manuscripts must include a data availability statement. This statement should provide the following information, where applicable:

- Accession codes, unique identifiers, or web links for publicly available datasets
- A description of any restrictions on data availability
- For clinical datasets or third party data, please ensure that the statement adheres to our policy

Data used in the study are available from the corresponding author upon reasonable request.

Human research participants

Policy information about studies involving human research participants and Sex and Gender in Research.

Reporting on sex and gender	This study was done on samples collected from participants of female sex.
Population characteristics	The samples were obtained from healthy participants aged 18-25 years old,
Recruitment	National Institute of Allergy and Infectious Diseases (NIAID), Vaccine Research Center (VRC) clinical trial at the National Institutes of Health (NIH) Clinical Center, Bethesda, Maryland, USA (ClinicalTrials.gov #NCT01132859).
Ethics oversight	The study was reviewed and approved by the NIAID Institutional Review Board and the Massachusetts General Hospital (MGH) institutional review board (IRB). The study team followed human experimental guidelines for conducting clinical research from the US Department of Health and Human Services.

Note that full information on the approval of the study protocol must also be provided in the manuscript.

Field-specific reporting

Please select the one below that is the best fit for your research. If you are not sure, read the appropriate sections before making your selection.

☒ Life sciences ☐ Behavioural & social sciences ☐ Ecological, evolutionary & environmental sciences

For a reference copy of the document with all sections, see nature.com/documents/nr-reporting-summary-flat.pdf

Life sciences study design

All studies must disclose on these points even when the disclosure is negative.

Sample size	Samples from 24 participants were used in this study.
Data exclusions	No data was excluded.
Replication	All experiments were performed at least in duplicate and all were successful.
Randomization	Participants were randomly selected to a 1:1 ratio.
Blinding	All experiments and data collection was done blinded.

Reporting for specific materials, systems and methods

We require information from authors about some types of materials, experimental systems and methods used in many studies. Here, indicate whether each material, system or method listed is relevant to your study. If you are not sure if a list item applies to your research, read the appropriate section before selecting a response.

Materials & experimental systems

n/a	Involved in the study	
☐	☒	Antibodies
☐	☒	Eukaryotic cell lines
☒	☐	Palaeontology and archaeology
☒	☐	Animals and other organisms
☐	☒	Clinical data
☒	☐	Dual use research of concern

Methods

n/a	Involved in the study	
☒	☐	ChIP-seq
☐	☒	Flow cytometry
☒	☐	MRI-based neuroimaging

Antibodies

Antibodies used	Pacific Blue conjugated mouse anti-human CD66b (BioLegend #305112, clone G10F5)<br>Fluorescein-conjugated goat anti-guinea pig complement C3 (MP Biomedicals, #0855385, polyclonal)<br>PE-Cy5 conjugated mouse anti-human CD107a (BD Biosciences, #555802)<br>PE-Cy7 conjugated mouse anti-human CD56 (BD Biosciences # 557747),<br>APC-Cy7 conjugated mouse anti-human CD16 (BD Bioscience #557758),<br>Pacific Blue conjugated mouse anti-human CD3 (BD Bioscience #558124),<br>Fluorescein-conjugated mouse anti-human Interferon gamma (BD Bioscience #550078)<br>PE conjugated mouse anti-human Macrophage Inflammatory Protein 1b (BD Bioscience 340449),<br>Mouse Anti-Human lgGl-PE (Southern-Biotech, #9054-09, clone:HP6001)<br>Mouse Anti-Human lgG2-PE (Southern-Biotech, #9060-09, clone:31-7-4)<br>Mouse Anti-Human lgG3-PE (Southern-Biotech, #9210-09, clone:HP6050)<br>Mouse Anti-Human lgG4-PE {Southern-Biotech, #9200-09, clone:HP6025)<br>Mouse Anti-Human lgM-PE (Southern-Biotech, #9020-09, clone:SA-DA4)<br>Mouse Anti-Human lgAl-PE (Southern-Biotech, #9130-09, clone: B3506B4)<br>Mouse Anti-Human lgA2-PE (Southern-Biotech, #9140-09, clone: A9604D2)
Validation	All antibodies are well established and quality controlled by the manufacturer. Additional information and references can be obtained on the company websites.<br>The use of antibodies 1-4 was previously validated: Brown EP, Licht AF, Dugast AS, Choi I, Bailey-Kellogg C, Alter G, et al. High-throughput, multiplexed lgG subclassing of antigen-specific antibodies from clinical samples. J lmmunol Methods. 2012;386(1-2):117-23.<br>Antibody 9 was described here: Fischinger, 5., J. K. Fallon, A. R. Michell, T. Brage, T. J. Suscovich, H. Streeck, and G. Alter. 2019. 'A high-throughput, bead-based, antigen-specific assay to assess the ability of antibodies to induce complement activation', J lmmunol Methods, 473: 112630.<br>Antibody 10: Karsten, C. B., N. Mehta, S. A. Shin, T. J. Diefenbach, M. D. Slein, W. Karpinski, E. B. Irvine, T. Brage, T. J. Suscovich, and G. Alter. 2019. 'A versatile high-throughput assay to characterize antibody-mediated neutrophil phagocytosis', J lmmunol Methods, 471: 46-56.

Eukaryotic cell lines

Policy information about cell lines and Sex and Gender in Research

Cell line source(s)	THP-1 human acute monocytic human cell line was acquired from ATCC.
Authentication	None of the cell lines used were authenticated.
Mycoplasma contamination	Cell line tested negative for Mycoplasma
Commonly misidentified lines<br>(See ICLAC register)	No commonly misidentified lines were used

Clinical data

Policy information about clinical studies

All manuscripts should comply with the ICMJE guidelines for publication of clinical research and a completed CONSORT checklist must be included with all submissions.

Clinical trial registration	NCT01132859
Study protocol	https://clinicaltrials.gov/ct2/show/NCT01132859
Data collection	The samples were collected under the Vaccine Research Center's (VRC), National Institute of Allergy and Infectious Diseases (NIAID), National Institutes of Health protocol VRC 900 (NCT01132859) in compliance with the NIH Institutional Review Board (IRB) approved protocol and procedures.
Outcomes	N/A

Flow Cytometry

Plots

Confirm that:

☒ The axis labels state the marker and fluorochrome used (e.g. CD4-FITC).

☒ The axis scales are clearly visible. Include numbers along axes only for bottom left plot of group (a 'group' is an analysis of identical markers).

☒ All plots are contour plots with outliers or pseudocolor plots.

☒ A numerical value for number of cells or percentage (with statistics) is provided.

Methodology

Sample preparation	Neutrophils were isolated from ACD blood using StemCell EasySep™ Direct Human Neutrophil Isolation Kit. Natural Killer cells (NK) were isolated from buffy coats using StemCell RosetteSep™ Human NK Cell Enrichment Cocktail
Instrument	Stratedigm 1300EXi , IntelliCyt® iQue Screener PLUS
Software	CellCapture 4<br>Forecyt 9
Cell population abundance	Neutrophils were identified through CD66b expression. Purity of neutrophils collected with isolation kit is usually >98%.<br>NK cells are identified by absence of CD3 expression and presence of CD16 and CD56 expression. Purity of NK cells collected with enrichment kit is usually >95%.
Gating strategy	All events were gated for granulocutes using FSC-H and SSC-H.<br>ADCP: Bead+ cells were identified as positive on the FITC channel.<br>ADNP: Neutrophils were selected for CD66b expression in the PB channel. ead+ cells were identified as positive on the FITC channel.<br>ADCD: Beads were gated on using FSC-H and SSC-H. Complement deposition was measured on the FITC channel as gMFI.<br>ADNKA: NK cells were identified through CD16 and CD56 expression as well as lack of CD3 expression. CD107a+ NK cells were identified by gating on the CD56+ cells on the PE-Cy5 channel. IFNg+ NK cells were identified by gating on the CD56+ cells on the FITC channel. The MIP1b+ cells were identified by gating on the CD56+ cells on the PE channel.

☒ Tick this box to confirm that a figure exemplifying the gating strategy is provided in the Supplementary Information.
